# Comparative evaluation of neonicotinoids and their metabolites-induced oxidative stress in carp primary leukocytes and CLC cells

**DOI:** 10.1038/s41598-024-59067-7

**Published:** 2024-04-09

**Authors:** Anna Rymuszka, Anna Sieroslawska

**Affiliations:** Department of Animal Physiology and Toxicology, Faculty of Medicine, The John Paul II Catholic University of Lublin, 1I Konstantynów Str., 20-708 Lublin, Poland

**Keywords:** Imidacloprid, Thiacloprid, Neonicotinoid metabolites, Desnitro-imidacloprid, Thiacloprid amide, Cytotoxicity, Cell biology, Immunology, Environmental sciences

## Abstract

Neonicotinoids (NEOs) have been designed to act selectively on insect nicotinic acetylcholine receptors (nAChRs). However, nAChRs are also expressed in vertebrate immune cells, so NEOs may interfere with the immune system in exposed non-target animals. The present study shows that NEOs: imidacloprid and thiacloprid, and their main metabolites: desnitro-imidacloprid and thiacloprid amide, at sub-micromolar concentrations ranging from 2.25 to 20 μM, affect the immune cells of fish. This was found both in primary cultures of leukocytes isolated from the carp head kidney and in the continuous adherent carp monocyte/macrophage cell line. Moreover, the results revealed that the studied pesticides and metabolites generate oxidative stress in carp immune cells and that this is one of the most important mechanisms of neonicotinoid immunotoxicity. Significant increases were observed in the formation of ROS and malondialdehyde (MDA). The antioxidant status alteration was linked with decrease in antioxidant enzyme activity: superoxide dismutase (SOD), catalase (CAT), and non-enzymatic antioxidant glutathione (GSH). Importantly, the metabolites: desnitro-imidacloprid and thiacloprid amide showed significantly higher cytotoxicity towards fish leukocytes than their parent compounds, imidacloprid and thiacloprid, which emphasizes the importance of including intermediate metabolites in toxicology studies.

## Introduction

Neonicotinoids (NEOs) are synthetic chemical substances used as plant protection products, characterized by a structure and action similar to nicotine. These substances were introduced to the market on a large scale in the mid-1990s, and since then, they have become the most commonly used pesticides in the world for the control of agricultural pests^1^. This is mainly due to their broad spectrum of activity and versatility in application. As systemic plant insecticides, they are used in arable farming and horticulture production to protect crops, such as grains and vegetables, against a range of pests. They also have veterinary applications, such as the control of ticks or fleas in pets^2^.

In comparison to other insecticides, such as pyrethroids, carbamates or organophosphorus compounds, which have a relatively short half-life, NEOs are characterized by an extended degradation period, enabling long-term protection of crops^1,3^. Specifically, imidacloprid (IMI) and thiacloprid (THI) are insecticides from the chloropyridinyl neonicotinoid chemical class that are characterized by persistence, which means that they have a relatively high ability to retain their physicochemical properties in the soil and exhibit significant bioconcentration capabilities^2,3^.

It should be emphasized that only about 5% of the active substance in neonicotinoid preparations is absorbed by the target crops; the majority is dispersed in the environment. NEOs and their metabolites are detected in various environmental compartments, including soil, surface water, as well as food: milk, fruit, vegetables and tea leaves^1,3,4^, which raises concerns about the safety of their use.

These substances are soluble in water, it is enough to apply a small amount of them to the seeds, and upon contact with water, they will dissolve and be absorbed by the roots of developing plants^5^. Observations showed that when seeds treated with IMI were repeatedly applied in a specific area, their concentration in soil samples increased significantly^6^. On the other hand, drift from spraying into water reservoirs and direct runoff to groundwater and then penetration into surface waters were observed. This is especially evident in areas with a low content of organic substances in the soil and steep slopes facilitating runoff from fields after heavy rainfall^7,8^. This manner of dispersion allows them to reach areas where they were not intentionally used^1,3,9^. Literature data indicate that NEO residues in the low ng/L range are often detected not only in surface waters, but also in drinking water in various countries around the world, e.g., Canada, the United States, Japan, Brazil, and China^4,10–13^. A meta-analysis on their concentrations in global waters, based on published data from several countries showed, that the mean concentrations of imidacloprid, nitenpyram, thiamethoxam, dinotefuran, imidaclothiz, acetamiprid, and thiacloprid were at levels of 119.54 ± 15.66 ng/L, 88.07 ± 27.14 ng/L, 59.75 ± 9.07 ng/L, 31.08 ± 9.27 ng/L, 24.54 ± 2.9 ng/L, 23.36 ± 4.01 ng/L, and 11.49 ± 5.09 ng/L, respectively^14^. Moreover, NEO residues are sometimes detected in every water sample tested^16^. There is limited information regarding the concentrations and dynamics of NEOs' degradation products and metabolites in the aquatic environment. In most cases, NEO metabolites are detected in water samples, but their amounts are near the detection limit or slightly above it. However, several monitoring studies from different regions of the world have found that the concentration of desnitro-imidacloprid (DN-IMI), the main metabolite of IMI, in water samples could be very high, ranging from tens (32 ng/L) up to about one hundred of ng/L (up to 106 ng/L)^15–18^.

NEO metabolites are typically detected in nanogram quantities, but the danger arises also from their stability and accumulative potential, which can sometimes be higher than for parent compounds as is the case with acetamiprid-N-desmethyl (AceND), the primary metabolite of acetamiprid^19^.

Many studies indicate that the continued presence of NEOs at low levels in soil, water, and wild plants may lead to negative impacts on various non-target organisms, including fish^3,7,9,20^. Negative effects on organs such as the gills, kidneys, brain, and liver have been observed in various fish species, including *Danio rerio*, *Prochilodus lineatus*, *Oreochromis niloticus*, *Gobiocypris rarus*, *Australoheros facetus*, *Astyanax altiparanae*, *Oncorhynchus mykiss* and *Cyprinus carpio* as a result of exposure to these compounds^21–28^.

The complex nature of exposure to many pesticides, which includes both parent compounds and numerous metabolites formed in the environment, complicates the toxicological assessment of these substances. It is known that NEOs are neurotoxins acting on the nicotinic acetylcholine receptor (nAChR). This receptor is located on the postsynaptic membrane and plays a fundamental role in neural signaling, regulating processes such as cell excitability and neuronal integration^1,4,6^. However, studies conducted in recent years have shown that nAChR is also expressed on immune cells, including lymphocytes, macrophages, mast cells, dendritic cells, and basophils and cholinergic transmission can modulate various aspects of both innate and adaptive immune response^29^.

It is worth noting that there is a lack of information regarding the effects of NEOs and their metabolites on fish defense mechanisms. In the literature, studies confirming the harmful effects on fish of IMI and THI, two NEO insecticides frequently detected in aquatic environments, involve pure parent compounds or pesticide formulations, not their metabolites. It is known that by generating DNA damage and inducing oxidative stress, they damage tissues of *Danio rerio*, *Oreochromis niloticus*, *Prochilodus lineatus*^22,23,27,30–33^.

In this study, we investigated whether IMI and THI, along with their main metabolites; DN-IMI and thiacloprid-amide (THI-A), pose potential cytotoxic and immunotoxic hazards. We assessed their impact on primary immune cells isolated from common carp (*Cyprinus carpio* L.) and fish cells from a stable cell line (CLC) obtained from carp monocytes. All experiments were independently performed three times.

## Materials and methods

### Test compounds

Insecticides: IMI, THI, and metabolite DN-IMI were purchased from Merck (Sigma Aldrich); THI-A was obtained from Dr. Ehrenstorfer Laboratory (Augsburg, Germany). Dimethyl sulfoxide (DMSO, Sigma Aldrich) was used to prepare stock solutions of tested compounds. Stock solutions were further diluted to the requested concentrations with phosphate-buffered saline (PBS, Sigma Aldrich). The final DMSO concentration in PBS solution was 0.1%.

### Cells line culture

The carp leukocyte cell line (CLC), which is a continuous adherent line of monocytes/macrophages, was purchased from the European Collection of Authenticated Cell Cultures (ECACC) and cultured in Eagle's Minimum Essential Medium (EMEM, Sigma Aldrich) with the following supplementation: 1% of non-essential amino acids (NEAA, Sigma Aldrich), 2 mM of L-glutamine, 100 IU and 0.1 mg/mL of penicillin/streptomycin solution (Sigma Aldrich), and 10% of fetal bovine serum (FBS, Sigma Aldrich), at 25 °C in a humid atmosphere containing 5% CO_2_. Cells at a stage of 80% confluence were used for further analysis.

### Isolation of primary leukocytes

Isolation of primary leukocytes for culture generally followed published protocols^34^. Head kidney (HK) tissue was removed from freshly slaughtered fish and placed into sterile containers with complete medium cRPMI (RPMI-1640 containing 100 IU and 0.1 mg/mL of penicillin/streptomycin solution, 1.5% of heat-inactivated pooled carp serum, supplemented with 10 IU mL of heparin; Sigma Aldrich). The research was carried out in accordance with the recommendations of directive no. 2010/63/EU and resolution 13/2016 of the National Ethics Committee for Animal Experiments of June 17, 2016. The approval of Ethic Committee was not required. Samples were collected from fish intended for commercial slaughter (breeding and slaughter farm, Poland).

The organs were homogenized in cRPMI solution using a steel sieve (100 µm nylon mesh, Sigma Aldrich), the resulting cell suspension was centrifuged at 300×*g* for 10 min and resuspended in 3.0 mL cRPMI. Then, the cell suspension was layered on a discontinuous Percoll gradient (1.06, 1.07, and 1.08 g/mL, Sigma Aldrich) and centrifuged for 30 min at 800×*g* at 4 °C. The fractions enriched in the different leukocyte subsets (1.020 g/mL—lymphocytes; 1.060 g/mL—monocytes/macrophages; 1.070 g/mL—granulocytes) were collected and washed three times. The composition of the isolated immune cell populations was analyzed by microscopic observation after cells were stained with May-Grünwald-Giemsa (Sigma Aldrich). Prior to further steps, cell number and viability were measured with the NucleoCounter YC-100 (Chemometec, Denmark), according to the manufacturer’s protocol.

### Experimental procedure

The concentrations of NEOs, and their metabolites used in the in vitro comparative studies were established by IC_50_ value (minimum concentration that gives 50% survival of cells) of IMI. To determine the IC_50_ of IMI, the CLC cells were incubated at 25 °C for 24 h with different concentrations of IMI ranging from 0 to 250 µM. The viability of cells was measured with MTT Assay (Roche, Sigma Aldrich) following the manufacturer's protocol. The IC_50_ value was calculated according to the dose–response curve of IMI generated using the software GraphPad Prism, and the IC_50_ value was determined on the level 78.5 ± 6.25 µM. To compare the toxic effects of selected NEOs and their metabolites, primary leukocytes and CLC cells were treated with the same concentrations and at the same time intervals. Tested concentrations of IMI, THI, and metabolites DN-IMI, and THI-A were 20 µM (1/5 IC_50_) and 10, 5, 2.5 µM. These concentrations were used to assess: the level of intracellular ATP, cell viability, reactive oxygen species production, antioxidant enzyme activities, and quantification of cellular glutathione. The exposure time to xenobiotics depended on the assay and was 3.5 h or 24 h. Cells without xenobiotic treatment served as the control group.

### Cytotoxicity and viability assays

Total cell ATP was measured using a CellTiter-Glo Luminescent Cell Viability Assay from Promega Corporation (Madison, WI, USA), according to the manufacturer’s directions. ATP level has been widely accepted as a valid marker of viable cells. When cells lose membrane integrity, they lose the ability to synthesize ATP and endogenous ATPases rapidly deplete any remaining ATP from the cytoplasm. For the study, primary leukocytes and CLC cells were seeded at 1 × 10^4^ cells per well in white 96-well plates and allowed to attach overnight in CO_2_ incubator at 25 °C. After cell adhesion, the medium was replaced with fresh medium without NEOs (control) or with NEOs or metabolites at 2.5, 5, 10, or 20 μM. Cells were further incubated for the next 24 h and then the CellTiter-Glo Reagent was added to all samples; the contents were mixed and incubated at room temperature for 30 min. Luminescence was quantified in the FLUOstar Omega plate reader (BMG Labortechnik, Germany). The results were expressed as the percentage of treated cells to untreated control cells, considered as 100%.

The effect of NEOs and their metabolites on the viability of fish immune cells was determined by using the same protocol of treatment as described above. The viability of cells was assayed using CellTiter-Blue Cell Viability Assay (Promega, Madison, WI, USA), containing a solution of resazurin. Viable cells with active metabolism reduce resazurin into the resorufin product which is pink and fluorescent. The quantity of resorufin produced is proportional to the number of viable cells, which was quantified using a microplate fluorometer equipped with a 560 nm excitation and 590 nm emission filter set. The resazurin solution was added directly to the cells that were incubated at a density of 1 × 10^5^ cells/well for 24 h in black 96-well plates with different concentrations of xenobiotics and to control cells. The supernatant fluorescence was subsequently quantified in the FLUOstar Optima multi-mode microplate reader. The results were expressed as the percentage of treated cells to untreated control cells, considered as 100%.

### Reactive oxygen species (ROS) quantitation

Oxidative stress was evaluated by the measurement of intracellular ROS production, level of hydrogen peroxide (H_2_O_2_), and lipid membrane alteration.

The generations of intracellular ROS in cells after treatment with NEOs or metabolites were determined by using the non-fluorescent probe, 20,70-dichlorodihydrofluorescein diacetate (DCFH-DA, OxiSelect Intracellular ROS Assay Kit, Cell Biolabs Inc., San Diego, CA, USA), that penetrates the cell membrane and is promptly oxidized by intracellular ROS forming the highly fluorescent product 2′,7′-dichlorodihydrofluorescein (DCF), which is easily detectable. The intensity of DCF fluorescence is proportional to the amount of ROS in the biological specimen. Briefly, fish leukocytes were placed at a density of 5 × 10^4^ cells per well in black 96-well plates and incubated under humid conditions at 25 °C, 5% CO_2_ overnight. After washing with PBS, cells were incubated with a DCF-DA probe in serum-free medium for 30 min. Thereafter, any residual DCFH-DA was eluted by the washing of PBS and leukocytes were resuspended in fresh PBS without NEOs (control) or with NEOs at 2.5, 5, 10, or 20 μM and incubated in the dark for another 3.5 h, during which the fluorescence intensity was measured at regular intervals (every 30 min) at an excitation wavelength of 480 nm and an emission wavelength of 530 nm by using the FLUOstar Optima multimode plate reader. The level of intracellular ROS was expressed by fluorescence intensity.

The H_2_O_2_ levels in leukocytes after exposure to NEOs were examined by ROS-Glo H_2_O_2_ Assay (Promega, Madison, WI, USA) according to manufacturer's guidelines. Cells (5 × 10^4^ cells/well) were cultured overnight in a white 96-well plate. On the next day, the cells were treated with different concentrations of studied compounds as described above. After 18 h incubation, the H_2_O_2_ Substrate was added to all wells and the plate returned to the incubator for the next 6 h. Then, the ROS-Glo Detection Solution was added to each well and the plate was left for incubation for 20 min at room temperature. The luminescence was measured using the plate reader. The results were expressed as the percentage of treated cells to untreated control cells considered 100%.

Lipid peroxidation was estimated based on the malondialdehyde (MDA) concentration, a final product of lipid peroxidation using a commercially available Lipid Peroxidation (MDA) Assay Kit (Sigma Aldrich), according to the manufacturer's instruction. Briefly, leukocytes (at a density of 1.0 × 10^6^ cells/well) were treated with the studied compounds as previously described. After 24 h incubation, cells were collected and homogenized on ice with MDA lysis buffer and then homogenates were centrifuged at 650×*g* for 15 min, 4 °C. Thiobarbituric acid (TBA) solution was added to all samples and heated in boiling water for 15 min. After cooling, the mixture was centrifuged at 650 g for 10 min, the supernatant was separated, and then absorbance was measured at 535 nm. The values of MDA in samples were calculated using MDA standards and expressed in nmol/mL.

### Preparation of cell lysate and detection of antioxidant enzyme activity

The activities of intracellular antioxidative enzymes, including superoxide dismutase (SOD) and catalase (CAT) was determined in the lysates of cells. Briefly, cells were cultured in the wells of a 24-well plate and treated with NEOs as described above. Following treatment with pesticides for 24 h, cells were solubilized in lysis buffer containing protease inhibitors for 30 min and sonication on ice. The cell lysates were centrifuged at 14,000×*g* for 5 min at 4 °C, then supernatants were aspirated and stored at -80 °C until measurement of CAT and SOD activity.

The activities of intracellular antioxidative enzymes were detected using the Superoxide Dismutase, SOD, Activity Assay Kit (Sigma-Aldrich), and Catalase Assay Kit (Sigma-Aldrich) according to the manufacturer’s protocol.

SOD activity in cells was determined by a biochemical method in which the inhibition of blue tetrazolium (NBT) reduction was measured using xanthine-xanthine oxidase as a superoxide generator. Briefly, standards, testing cell lysates, WST (tetrazolium salt) working solution, and xanthine oxidase working solution were placed into a 96-well microplate. After 30 min of incubation at room temperature, the enzyme inhibition activity was determined spectrophotometrically (440 nm).

CAT is an antioxidant enzyme that catalyzes the breakdown of H_2_O_2_ into water and oxygen. CAT activity was measured by monitoring the rate of H_2_O_2_ decomposition over a specific period of time. Samples (cell supernatants) were added to microtubes containing Assay Buffer (50 mM potassium phosphate buffer), and incubated with H_2_O_2_ (200 mM) for 1 min. The reaction was stopped, and the reaction mixture was transferred to cuvettes and chromogen was added. CAT activity was determined by measuring the absorbance at 540 nm using the FLUOstar Optima plate reader.

### Detection of glutathione levels

The levels of intracellular glutathione reductase (GSH), oxidized (GSSG) glutathione and their ratio (GSH/GSSG) were measured according to the instructions of the Promega test kits:

The GSH-Glo glutathione assay kit is based on the conversion of a luciferin derivative to luciferin in the presence of glutathione, catalyzed by glutathione S–S-transferase (GST). Briefly, leukocytes were plated in a white 96-well microplate and allowed to attach overnight in a CO_2_ incubator at 22 °C; cells were then washed with PBS and exposed to various concentrations of NEOs or metabolites (each determination was performed in triplicate) for another 24 h. The cell medium was then removed, GSH-Glo reagent was added to the cells and incubated at room temperature for 30 min. The reconstituted Luciferin Detection Reagent was then added to each well and the luminescence was measured using the multimode microplate reader.

To determine both total GSH and oxidized glutathione (GSSG) and to calculate the GSH/GSSG ratio in treated cells, a similar approach was used using the GSH/GSSG-Glo Glutathione Assay. Primary leukocytes and CLC cells (1 × 10^5^ cells/mL) were cultured and exposed to NEOs as described above. After incubation of the cells with studied compounds, the medium was removed and replaced with either Total Glutathione Lysis Reagent or Oxidized Glutathione Lysis Reagent and mixed briefly using the plate shaker. Next, Luciferin Generation Reagent was added to the samples; the plates were incubated for 30 min at room temperature. After shaking, luminescence was measured using the FLUOstar Omega plate reader. A standard curve for GSH concentration was generated and the results were expressed as either concentrations of GSH, or GSSG, and the GSH/GSSG ratio was determined according to the manufacturer's guidelines.

### Statistical analysis

Significant differences in biological parameters between the treatment and control groups were evaluated using a one-way variance (ANOVA) followed by Tukey’s test. All analyses were performed using Statistica 10.0 software (StatSoft Inc., Tulsa, OK, USA). The results were considered statistically significant at p < 0.05.

## Results

### Selected NEOs and metabolites slightly reduce CLC cells and HK leukocyte viability

**Figure 1:**
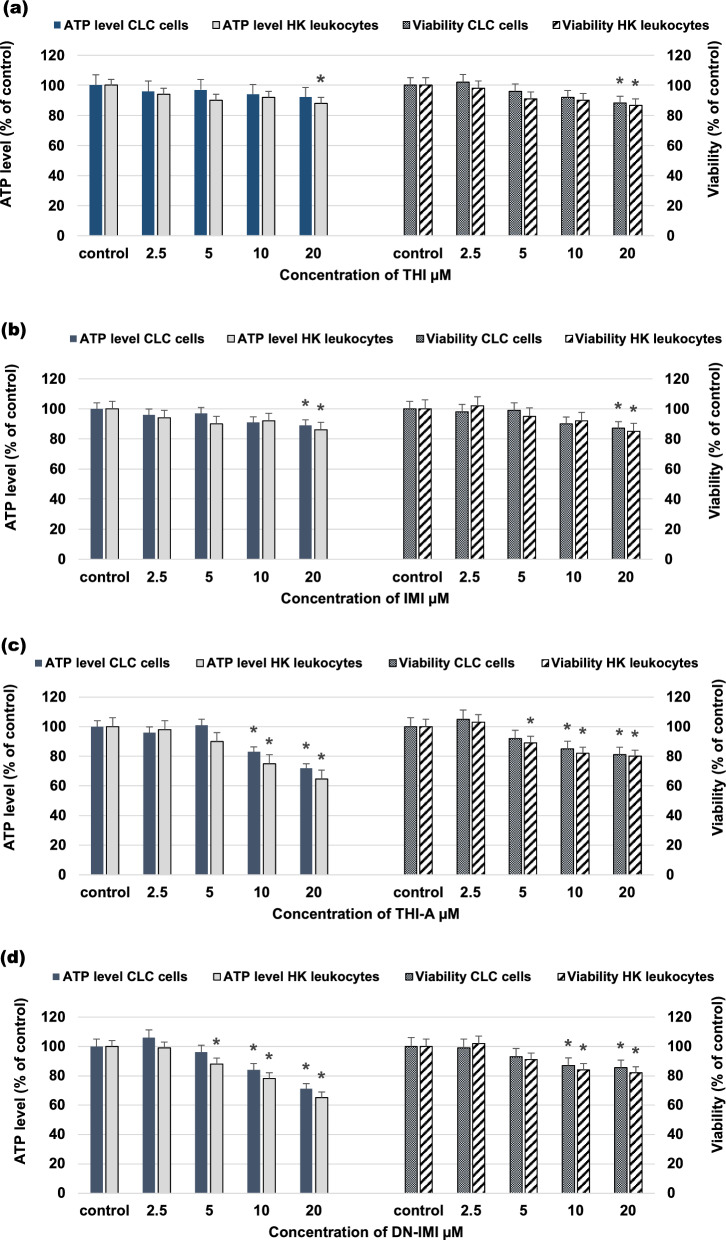
Viability and ATP level in the carp HK leukocyte and CLC cells exposed to different concentrations ranging from 2.25 to 20 μM of thiacloprid (THI; **a**), imidacloprid (IMI; **b**) and their main metabolites: thiacloprid amide (THI-A; **c**) and desnitro-imidacloprid (DN-IMI; **d**) for 24 h. Values are expressed as mean ± SE. The asterisk represents a statistical difference at p˂ 0.05.

The cytotoxicity of the THI, IMI, and metabolites: THI-A, DN-IMI at different concentrations ranging from 2.25 to 20 μM on fish primary leukocytes and CLC cells was determined (Fig. 1). At the highest concentration used of the parent compounds (20 μM), cell viability was on the level 88% (CLC cells) and 87% (HK leukocytes) for THI and 87% (CLC cells) and 85% (HK leukocytes) for IMI, respectively. Compared to the control, this was a statistically significant decrease in cell viability. Likewise, quantitation of ATP based on the number of metabolically active cells showed that 92% and 89% of CLC cells; and 89% and 86% of the primary HK cells remained viable when treated with THI and IMI, respectively. No visible decrease in cell viability was observed at concentrations of parent NEO compounds lower than 20 μM (≥ 90% viability). Noteworthy, the original compounds did not affect the cell viability that much and possessed negligible toxicity. While for the cytotoxicity assessment of the main metabolites of these insecticides at the 20 μM, cell viability dropped to 84% (CLC cells) and 83% (HK leukocytes) for THI-A and 85% (CLC cells) and 82% (HK leukocytes) for metabolite DN-IMI. Further, when studying ATP levels at 20 μM, the survival percentage of THI-A and DN-IMI treated cells was only about 72% (CLC cells) and 65% (HK leukocytes), respectively. The results showed that the cytotoxicity of the metabolites was slightly higher than that of the original compounds. The metabolites at a concentration of 10 µM caused a statistically significant decrease in viability and ATP levels, while the original compounds at the same concentration did not decline these parameters.

### NEOs and metabolites generated the formation of excess ROS and induced oxidative damage

**Figure 2:**
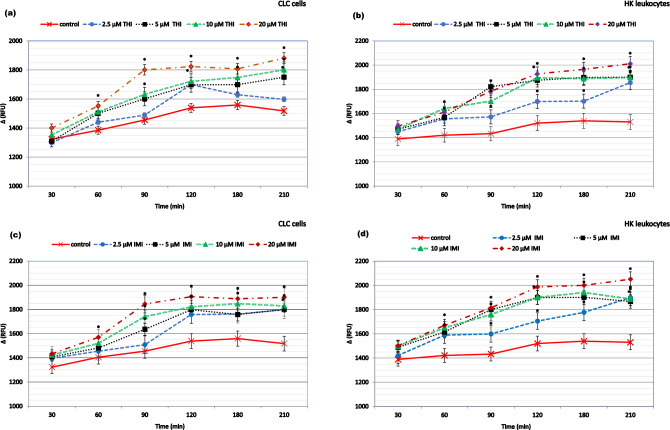
The reactive oxygen species (ROS) production in carp HK leukocytes and CLC cells exposed to different concentrations ranging from 2.25 to 20 μM of thiacloprid (THI; **a**,**b**), imidacloprid (IMI; **c**,**d**) for a period from 30 min. to 3.5 h. Values are expressed as mean ± SE. The asterisk represents a statistical difference at p˂ 0.05.

**Figure 3:**
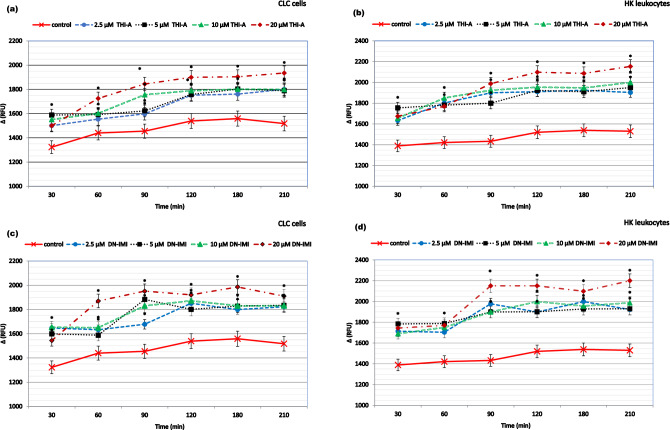
The reactive oxygen species (ROS) production in carp HK leukocytes and CLC cells exposed to different concentrations ranging from 2.25 to 20 μM of metabolites: thiacloprid amide (THI-A; **a**,**b**) and desnitro-imidacloprid (DN-IMI; **c**,**d**) for a period from 30 min. to 3.5 h. Values are expressed as mean ± SE. The asterisk represents a statistical difference at p˂ 0.05.

The formation of excess hydroxyl, peroxyl, or other reactive oxygen species activity within primary cell cultures and CLC cells after exposure to the NEOs and their metabolites is shown in Figs. 2 and 3, respectively. During cell culture maintenance, the level of ROS content fluctuates. This is not a constant parameter, as cells can either proliferate or undergo spontaneous apoptosis. Therefore, the kinetics of changes in this parameter within the studied groups were compared relative to the ROS content in the control group over the same time interval. It was found that all compounds studied in concentrations ranging from 2.5 to 20 µM caused an increase in ROS levels within the cell cytosol, compared to the changes observed in the control group in the same time intervals. Moreover, cells showed different sensitivity to their pro-oxidant effects. More specifically, the primary cell cultures were more sensitive to the tested xenobiotics than CLC cells. Notably, elevated ROS levels in compounds-exposed leukocytes were evident, but not clearly dose-dependent. Exposure of cells to NEOs and metabolites increased ROS production, which peaked at 1.5–2 h and remained elevated for the duration of the experiment.

**Figure 4:**
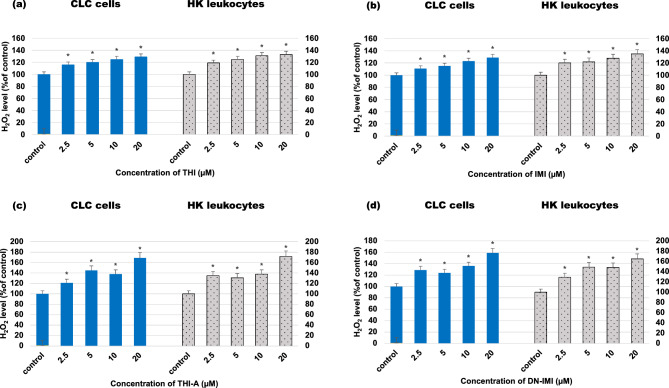
Hydrogen peroxide (H_2_O_2_) level in carp HK leukocytes and CLC cells exposed to different concentrations ranging from 2.25 to 20 μM of thiacloprid (THI; **a**), imidacloprid (IMI; **b**) and their main metabolites: thiacloprid amide (THI-A; **c**) and desnitro-imidacloprid (DN-IMI; **d**) for 24 h. Values are expressed as mean ± SE. The asterisk represents a statistical difference at p˂ 0.05.

Since H_2_O_2_ is the most stable and has the longest half-life of all ROS and in addition various ROS are converted to H_2_O_2_ within cells (e.g., SOD converts superoxide to O_2_ and H_2_O_2_), we also measured the relative level of H_2_O_2_ directly in the cell culture of leukocytes after 24 h incubation with NEOs and their main metabolites (Fig. 4). Luminescent signal, proportional to H_2_O_2_ concentration, was positively correlated with the concentration of compound being tested. The results showed that 24 h treatment of CLC cells with 2.5, 5, 10, and 20 μM THI stimulated the intracellular ROS generation by about 16%, 20%, 27%, and 29% respectively, compared to control; and incubation HK leukocytes caused even higher increase—by about 20%, 25%, 31%, 33%, respectively. In both tested types of cells, a similar increase in ROS generation was noticed, also after exposure to IMI. Especially high level of ROS production has been observed in the case of leukocyte exposure to neonicotinoid metabolites The study showed even a 69% and 59% increase in H_2_O_2_ production in response to the exposure of CLC cells to the highest concentration (20 µM) of THI-A and DN-IMI, and 72% and 65% augmentation of cellular ROS generation in HK leukocytes, appropriate. Moreover, a significant increase in H2O2 production was observed in cells exposed to lower concentrations of metabolites, although no clear concentration-dependent effect was observed, as in the case of incubation of cells with parent compounds.

**Figure 5:**
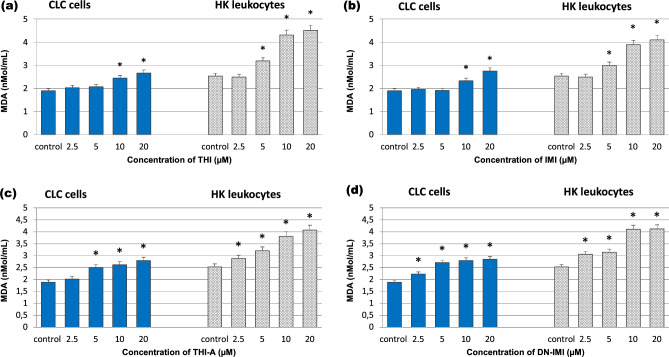
Lipid peroxidation (LPO) level in carp HK leukocytes and CLC cells exposed to different concentrations ranging from 2.25 to 20 μM of thiacloprid (THI; **a**), imidacloprid (IMI; **b**) and their main metabolites: thiacloprid amide (THI-A; **c**) and desnitro-imidacloprid (DN-IMI; **d**) for 24 h. Values are expressed as mean ± SE. The asterisk represents a statistical difference at p˂ 0.05.

Lipid peroxidation (LPO) is cellular oxidative damage to membrane lipids that is recognized as an important and useful marker of oxidative stress. LPO can be measured by evaluating the level of end products (such as MDA) of peroxidation of polyunsaturated fatty acids susceptible to ROS attack. Figure 5 shows that both the original NEOs and their main metabolites change the MDA contents in leukocytes; however, they generate different levels of oxidative damage in the membrane lipids of incubated cells. It was also noted that original compounds caused smaller changes in the parameter studied compared to metabolites. In particular, treatment of CLC cells with the lower (2.5 and 5 µM) concentrations of THI and IMI did not significantly increase the MDA levels; however, treatments with 10, and 20 µM led to a significant (29% and 41% for THI, and 23% and 45% for IMI, respectively) increase in MDA contents as compared to control groups (Fig. 5a,b). Interestingly, the original compounds of neonicotinoid even at 5 µM caused a statistically significant increase in the MDA level in HK leukocytes, confirming the higher sensitivity of these cells to the NEOs. It was also noted that original compounds caused smaller changes in the parameter studied compared to their metabolites.

As shown in Fig. 5c,d, MDA levels were increased in both cell cultures exposed to studied metabolites of NEOs at all assayed concentrations from 11 to 48% for THI-A, and from 18 to 51% for DN-IMI in case CLC cells; and from 14 to 61% for THI-A and from 21 to 63% for DN-IMI in case primary leukocytes.

### NEOs and metabolites altered the redox status in CLC cells and primary leukocyte culture

**Figure 6:**
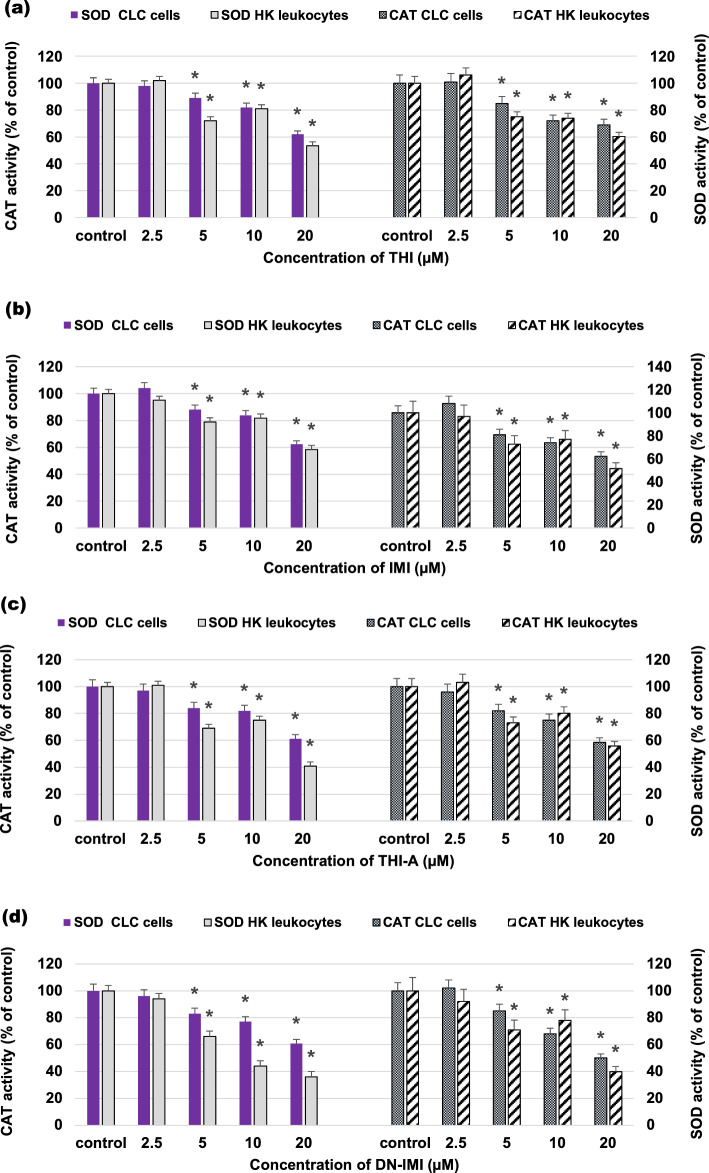
The antioxidant enzymes superoxide dismutase (SOD) and catalase (CAT) activity in carp HK leukocytes and CLC cells exposed to different concentrations ranging from 2.25 to 20 μM of thiacloprid (THI; **a**), imidacloprid (IMI; **b**) and their main metabolites: thiacloprid amide (THI-A; **c**) and desnitro-imidacloprid (DN-IMI; **d**) for 24 h. Values are expressed as mean ± SE. The asterisk represents a statistical difference at p˂ 0.05.

The redox status is regulated by a careful balance between the generation of ROS and their elimination. The antioxidant enzymes SOD and CAT are the body's first line of defense against ROS. As shown in Fig. 6a–d, the specific activities of SOD, and CAT, were at different levels altered in cells treated with the original NEOs (THI, IMI) and their metabolites (THI-A, DN-IMI) compared to the control. The significant decline in the activity of antioxidant enzymes was observed in the leukocytes incubated with the higher concentrations (5, 10, and 20 µM) of tested compounds. A differential decrease in enzyme activity was noticed in relation to different concentrations of THI and IMI and metabolites (THI-A and DN-IMI) in primary cell culture. The level of inhibition of SOD activity in the presence of the highest concentrations of compounds was the largest and almost identical in CLC cells (38% for THI; 37% for IMI; 39% for THI-A; and 38% for DN-IMI), while the inhibition of SOD activity in exposition HK leukocytes was 46% for THI, 41% for IMI, 59% for THI-A and 64% for DN-IMI, respectively. Similarly, a decline and the highest significant difference in the CAT activity were observed between control and compound-treated cells at 20 μM. THI and IMI caused approximately 30% and 38% reduction of the parameter studied in the CLC cells, and 40% and 48% in the HK leukocytes, respectively. While THI-A and DN-IMI declined in CAT activity in exposed CLC cells by approximately 42% and 50% and 44% and 60% in primary cells. It is worth mentioning that the activity of SOD and CAT decreased with the increase in NEOs and metabolite concentrations in case of CLC cells, but not HK leukocytes. This differential response may be due to the presence of other cell types in primary leukocyte cultures that can alter either the level of enzymes or modulate their activity.

**Figure 7:**
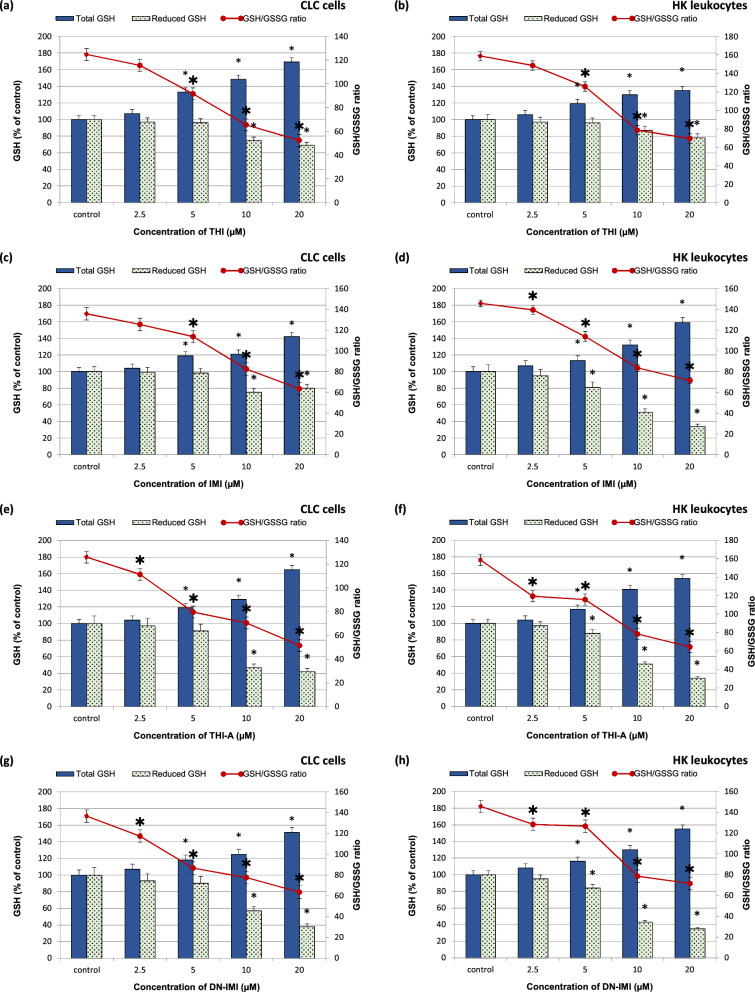
The total and reduced glutathione level (GSH) and the reduced oxidized glutathione ratio (GSH/GSSG) in carp HK leukocytes and CLC cells exposed to different concentrations ranging from 2.25 to 20 μM of thiacloprid (THI; **a**,**b**), imidacloprid (IMI; **c**,**d**) and their main metabolites: thiacloprid amide (THI-A; **e**,**f**) and desnitro-imidacloprid (DN-IMI; **g**,**h**) for 24 h. Values are expressed as mean ± SE. The asterisk represents a statistical difference at p˂ 0.05.

It is well known that glutathione is one of the major nonenzymatic components of intracellular antioxidant defenses that protect the cell against the harmful toxic effects of xenobiotics. Intracellular glutathione usually exists in its reduced form (GSH), but in some conditions (by interactions with ROS/RNS) it can be converted into the oxidized form, GSSG, this form is accumulated, which changes the ratio of GSH to GSSG. Therefore, the redox status of cells is often evaluated by measuring the concentrations of reduced glutathione (GSH), oxidized (GSSG) glutathione, and their ratio (GSH/GSSG). The results have shown that the original compounds of NEOs and their main metabolites in all concentrations used caused a decrease in the content of reduced GSH in both exposed cell cultures relative to the control cells (Fig. 7).

In particular, a significant and concentration-dependent decrease in GSH content in metabolites treated cells was observed. THI-A at the highest concentrations (10 µM and 20 µM) caused approximately 53% and 58% reduction of the parameter studied in the CLC cells, and 49% and 66% in the HK leukocytes, respectively (Fig. 7c,d). Likewise, a comparable effect was observed in cells treated with DN-IMI. At the 10 µM and 20 µM of the metabolite declined in GSH content in exposed CLC cells by approximately 43% and 62% and 57% and 65% in primary cells was detected.

In parallel, oxidized glutathione (GSSG) content was measured and its significant increase was observed in treated cells (Fig. 7). After incubation of the cells with the studied original NEOs and their main metabolites at 2.5 μM, there was no significant increase in cellular GSSG, but GSSG content was statistically increased at the concentrations ≥ 5 μM.

A decline in GSH content in exposed cells clearly indicates the disturbance in their antioxidant defense system as also confirmed by analysis of the ratio of reduced to oxidized glutathione (GSH/GSSG). The GSH/GSSG ratios in treated cells declined in a concentration-dependent manner, but no significant difference was observed between cell cultures treated with the same concentrations of compounds. In the case of CLC cell exposure to the highest concentration (20 µM) of THI and IMI, it was observed that the values of the examined parameter in the control cells compared to the cells in the exposed group decreased from 125 to 52 and from 135 to 63, respectively. Meanwhile, in HK leukocytes, the GSH/GSSG ratio decreased from 158 to 69 and from 145 to 71, respectively. A similar trend in cells exposed to metabolites was also observed.

## Discussion

The present study aimed to assess the impact of sublethal concentrations of IMI and THI, as well as their main metabolites (DM-IMI and THI-A), on primary immune cells isolated from the head kidney of carp and on continuous CLC carp cell line. To our knowledge, this is the first analysis that compares the sensitivity of carp leukocytes to both parent compounds and the major metabolites of NEOs under established conditions.

According to the obtained results, IMI and THI did not significantly reduce the viability of carp primary leukocytes or CLC cells at any of the tested sublethal concentrations (≥ 85% viability). The cytotoxicity of the metabolites was slightly higher than that of the original compounds, but only at the highest concentration (20 µM) causing the drop of the survival of fish leukocytes to about 70%. Similarly, Walderdorffa et al.^35^ compared the influence of IMI on invertebrate and vertebrate immune cells and showed that the viability of human macrophages (THP-1) and *Drosophila melanogaster* hemocytes (Schneider 2 cells) after exposure to IMI was not significantly decreased but at the same time, they observed an adverse effect on the phagocytic activity in both types of immune cells. Moreover, IMI had a stronger immunosuppressive effect on invertebrate cells than mammalian phagocytes.

It is well documented that NEOs have a stronger neurotoxic effect on invertebrate cells than vertebrate cells. IMI causes neural Kenyon cell death in the cockroach *Periplaneta americana* and decreases cell viability following compound exposure to concentrations higher than 50 μM^36^. The EC_50_ value for honey bee Kenyon cells was estimated at approximately 6.3 μg/mL (24 μM) of IMI^37^, whereas for mouse immortal neuronal cell lines Neuro-2a the IC_50_ has been determined at the level of 1152.1 μM^38^.

Over the last decade, several studies reporting effects on the survival, morphological responses, and functioning of different types of cells after exposure to NEOs have been published. Interestingly, there are significant differences in the sensitivity of individual cells to the same pesticides. Cell viability and IC_50_ values depend on the cell and tissue type, as well as the species from which they originated. In studies performed by Abdel-Halim^39^, the IC_50_ value of IMI was only 25 μM for the human prostate epithelial WPM-Y.1 cell line. A recent study by Silva et al.^40^, comparing the cytotoxic effects of IMI on six different cell lines, namely: Caco-2 (human epithelial colorectal adenocarcinoma), HepG2 (human hepatocellular carcinoma), A431 (human epidermoid carcinoma), HaCaT (human keratinocytes), SK-MEL-5 (human skin melanoma), and RAW 264.7 (mouse macrophages), demonstrated the highest sensitivity of immune cells to this insecticide. The IC_50_ value was approximately 1000 μM for Caco-2 cells; 623.8 μM for HepG2 cells; 573.2 μM for A431 cells; 506.6 μM for SK-MEL-5 cells; 495.3 μM for HaCaT cells, while for RAW 264.7 cells the IC_50_ was 305.9 μM. In the present experimental setup, the IC_50_ value of IMI for fish immune cells (CLC) was 78.5 μM.

Generally, it is presumed that the modulating effect of xenobiotics on the immune response occurs through their interaction with mechanisms that control the production of reactive oxygen and nitrogen species (RONS)^41^. Taking this into consideration, the present study aimed to comparatively evaluate the impact of selected NEOs and their major metabolites on the redox state of carp immune cells. It is known that oxidative changes are a natural consequence of cellular metabolism resulting from aerobic respiration and the generation of RONS^42^. The activation of immune cells requires increased energy demand obtained at the expense of aerobic metabolism. Physiologically released RONS act as mediators and regulators, ensuring the proper functioning of immune cells. Redox mechanisms regulate and modulate various immune functions, including metabolic reprogramming of immune cells, antigen recognition, polarization of macrophages and Th lymphocytes, cytokine production, pathogen detection, chemotaxis, and phagocytosis^42^.

Oxidative stress induced in immune cells by cytokines or growth factors under physiological conditions is typically short-lasting and not harmful to these cells. The redox balance of cells is quickly restored by antioxidant systems. However, when the activation of oxidative processes exceeds the compensatory capacity of the cell, and the duration of oxidative stress is prolonged, pathological changes occur within the cell. Such a situation arises when external factors, such as pesticides, contribute to an increased production of RONS on one hand and a decrease in the cell's antioxidant potential on the other hand. In this study, it was found that exposure of leukocytes to both parent substances and metabolites at the concentrations, which did not cause a visible decrease in cell viability, significantly affected the redox state in the cells, causing a significant increase in the level of ROS both in primary cultures and in cells of the continuous line. The antioxidant status alteration was linked with dysfunction of antioxidant enzymes activity: SOD, CAT, and non-enzymatic antioxidant GSH, an increase in the ratio of GSH/GSSG, which is a good indicator of cellular oxidative stress, and lipid peroxidation.

These results are in accordance with previous in vitro experiments, where NEOs have been noted as chemical stressors that induce severe shifts in different types of cells by oxidative damage^43,44^. Studies performed on Caco 2 cells, which are a model of the intestinal epithelium, reported that IMI induced a significant increase in ROS content, and it affects intestinal barrier functions through the increase of proinflammatory cytokine production and decreases in adhesiveness of enterocytes^43^. Silva et al.^40^ confirmed the cytotoxic effects of IMI on Caco-2 and HepG2 cells, the increase in lipid peroxidation, and the increase in GSH content, most likely in response to the increase in ROS content. Another study conducted by Abdel-Halim and Osman^39^ indicated that IMI caused in vitro cytotoxic and oxidative stress on normal human cells (prostate epithelial WPM-Y.1 cell line). They found that exposure to IMI significantly reduced cell viability and induced cytotoxicity characterized by cell death in the tested cells, alterations of oxidative stress enzyme activity, increased levels of LPO, and LDH activity as well as histopathological alterations.

The incubation of Chinese hamster ovary (CHOK1) cells with IMI led to genotoxic effects with significant inhibition of the activity of GST, glutathione peroxidase (GPx), and glutathione reductase (GR)^44^. In contrast, Guo et al.^45^ showed that IMI may induce gene mutation, chromosome breakage, and DNA damage in human lymphoblastoid TK6 cells, but according to the authors, the formation of ROS does not seem to be involved in a mechanism of genotoxicity. Similarly, no significant ROS generation was found after exposure to IMI in human Jurkat cells, but increased DNA damage and higher frequencies of micronuclei (MN) and sister chromatid exchange were observed^46^.

Some in vitro studies have also suggested that the overproduction of ROS in intracellular space is involved in the genotoxicity of THI. Galdikova et al.^47^ demonstrated that incubation of bovine peripheral lymphocytes with THI induces oxidative DNA damage, leading to unstable chromosome aberrations and dysregulation of the expression of bovine glutathione S–S-transferase M3 (GSTM3). Moreover, genetic instability decreased cell viability and proliferation, and apoptosis was probably mediated by increased levels of oxidative damage in bovine peripheral lymphocytes exposed to THI^48^. A similar recent study by Parny et al.^49^ reported that THI promoted the ability of human monocyte-derived macrophages (hMDMs) to produce ROS in response to zymosan and induced the production of IL-10 in response to lipopolysaccharide (LPS) that may affect immune homeostasis.

Generally, many studies indicate that oxidative stress induced by IMI and THI at the molecular level causes DNA damage and apoptosis, which leads to histopathological changes in various fish organs, resulting in developmental abnormalities, neurotoxicity, hepato-renal toxicity, endocrine system disorders, hematological changes, and immunosuppression^20–25,27,28^.

One important area where further research is needed is the assessment of the potential toxicity of metabolites formed during the biotransformation of NEOs, which are found in trace amounts in samples of water, soil, food, and biological material^1,8,9,50–52^. Usually, the modified bioactivity is a result of the transformation of the electron-rich nitro- or cyano- functional groups found in the original neonicotinoid compound. Importantly, there are differences in the transformation of the parent compounds and the accumulation of individual neonicotinoid metabolites in various components of the environment. DN-IMI is the main degradation product of IMI in the environment, and 5-hydroxy-imidacloprid (5-OH-IMI) is a characteristic metabolite of IMI in mammals and plants^1–3^. Moreover, there are many uncertainties regarding the metabolic conversion that NEOs undergo in the body of various animal species. For example, in the mammalian liver, IMI by cytochrome p450 monooxygenases (CYPs), various pathways such as hydroxylation, N-demethylation, and nitroimine reduction^2,3^, is converted into many different metabolites. Recent studies confirm the presence in human matrices of metabolites with varying degrees of toxicity, including DN-IMI, 5-OH-IMI, and imidacloprid-olefin (IMI-olefin)^52–55^.

Moreover, sometimes intermediate metabolites are not only more toxic, but also more persistent than the parent compound, which will increase the possibility of their bioconcentration in the soil environment^56^, and thus the risk of penetrating into surface waters which poses a potential threat to aquatic organisms. For example, the half-life of THI in laboratory and field conditions ranges from 5 to 27 days^57^, while its major metabolite THI-A ranges from 32 to 142 days in laboratory soil and from 46 to 314 days in field soil^58^.

The present study shows that the metabolites DN-IMI, and THI-A at non-cytotoxic concentrations affect fish leukocytes. It was found that after exposure in cells of both primary and continuous cultures, the level of production of H_2_O_2_ and other reactive oxygen species increases significantly. In addition, there is a significant dysregulation and decrease in the activity of SOD and CAT enzymes and a reduction in the antioxidant defense capacity of immune cells, as evidenced by the increased GSH/GSSG ratio and lipid peroxidation. Studies clearly indicate that both DN-IMI and the metabolite THI-A disturb the homeostasis of fish immune cells to a greater extent than the parent substances.

To date, there have been few studies on the toxicity of IMI metabolites, but they indicate that DN-IMI may be significantly more toxic to vertebrates than the parent substance of IMI. This metabolite has a documented higher affinity for mammalian or chicken nAChRs^59–61^. Loser et al.^62^ showed that DN-IMI at sub-micromolar concentrations is equipotent to nicotine and can be several orders of magnitude more potent as a neuronal signaling disrupter than its parent compound. Mourikes et al.^63^ showed that DN-IMI affects mouse ovarian antral follicle growth, morphology, and hormone synthesis in vitro. Metabolite inhibited follicle growth and caused follicles to rupture in culture, decreased progesterone, testosterone, and estradiol levels and dysregulated the expression of steroidogenic regulators, estrogen receptors, and apoptotic factors.

Recent bioinformatic analysis carried out by Sinclair et al.^64^ indicates that the exposure of LUHMES cells to IMI, and its metabolites: DN-IMI, and IMI-olefin has been found to significantly impact proteins associated with signal transduction, genomic transcription, protein translation, and RNA-related processes, such as RNA binding and ribosomal networks. The authors suggest that such a proteomic analysis may be useful in understanding the complex mechanisms of cellular responses involved in the development of long-term neurotoxicity.

Although THI is also a common NEO in water, soil, and biological samples, its enzymatic biotransformation and toxicity of metabolites are poorly understood. Studies on vertebrates, including rats, mice, and chickens, have shown that THI after being taken into the body, is intensively metabolized to over 30 different metabolites, including: olefin, imine, amide, sulfoxide and hydroxythiazolidine^65^.

Importantly, these metabolites are assumed to have similar carcinogenic properties and toxicity as THI, which has been classified as a category 2 carcinogen according to the regulation of the European Parliament and labeled as potentially toxic for reproduction in humans^66,67^.

Nevertheless, structural modifications of the parent compound affect the bioactive potential of the resulting metabolites and their degree of toxicity for various species to a varying degree. Toxicological studies have shown that THI-A has a stronger toxic effect on the soil microbial community than THI, but is less toxic to aquatic invertebrate *Daphnia magna*^68^.

Recent studies by Serrano et al.^69^ have confirmed that THI can undergo intensive biotransformation in the liver of fish by different paths: reductive decyanation, reductive dechlorination with hydration, and dealkylation processes, resulting in three primary metabolites: TM1, TM2 (thiacloprid—amine), and TM3.

The present study shows that THI-A, like DN-IMI, is more damaging to carp leukocytes than the parent substance. Exposure of immune cells to metabolites disturbs the redox state of these cells already at the lowest concentration tested, 2.5 µM.

The primary mechanism of THI action follows the general toxicity pathway of IMI with disruption of the organism’s nervous system by stimulation of nicotinic acetylcholine receptors responsible for fast neurotransmission. It should be noted that immune cells possess an operational cholinergic system, and nAChRs play a role in controlling T cell differentiation, as well as the production of antibodies specific to antigens and proinflammatory cytokines. For this reason, it seems to be very important to conduct further studies on the effects of neonicotinoid metabolites on immune cells to document and understand correlations between changes in oxidative biomarkers and immunotoxicity and to link the risk of exposure to biologically relevant consequences for animal health.

## Conclusions

NEOs have been designed to act selectively on the nAChRs of insects. Nevertheless, nAChRs are also expressed in vertebrate immune cells, and NEOs may interfere with immune function in exposed non-target animals. The present study shows that NEOs: IMI and THI, and their main metabolites DN-IMI and THI-A, potently (at sub-micromolar concentrations) affect fish immune cells. This was found both in primary cultures and in the continuous adherent carp monocyte/macrophage line. Moreover, the results revealed that the studied pesticides and metabolites generate oxidative stress in carp immune cells and that this is one of the most important mechanisms of neonicotinoid immunotoxicity. Significant changes were observed in the formation of excess ROS, SOD and CAT activity, GSH levels, and MDA contents. Importantly, the metabolites DN-IMI and THI-A exhibited significantly higher cytotoxicity potency on fish leukocytes than their parent compounds IMI and THI, which emphasizes the importance of including intermediate metabolites in toxicology studies and risk assessment.

## Data Availability

All data generated or analyzed during this study are included in this paper. Any additional data or subset of datasets used for this study are available from the corresponding author upon request.
